# Optimal design of cluster randomized trials allowing unequal allocation of clusters and unequal cluster size between arms

**DOI:** 10.1002/sim.9135

**Published:** 2021-07-27

**Authors:** Andrew J. Copas, Richard Hooper

**Affiliations:** 1 MRC Clinical Trials Unit, University College London, London, UK; 2 Centre for Primary Care and Public Health, Queen Mary University of London, London, UK

**Keywords:** cluster randomized trial, optimal design, power, sample size, unequal allocation

## Abstract

There are sometimes cost, scientific, or logistical reasons to allocate individuals unequally in an individually randomized trial. In cluster randomized trials we can allocate clusters unequally and/or allow different cluster size between trial arms. We consider parallel group designs with a continuous outcome, and optimal designs that require the smallest number of individuals to be measured given the number of clusters. Previous authors have derived the optimal allocation ratio for clusters under different variance and/or intracluster correlations (ICCs) between arms, allowing different but prespecified cluster sizes by arm. We derive closed-form expressions to identify the optimal proportions of clusters and of individuals measured for each arm, thereby defining optimal cluster sizes, when cluster size can be chosen freely. When ICCs differ between arms but the variance is equal, the optimal design allocates more than half the clusters to the arm with the higher ICC, but (typically only slightly) less than half the individuals and hence a smaller cluster size. We also describe optimal design under constraints on the number of clusters or cluster size in one or both arms. This methodology allows trialists to consider a range for the number of clusters in the trial and for each to identify the optimal design. Except if there is clear prior evidence for the ICC and variance by arm, a range of values will need to be considered. Researchers should choose a design with adequate power across the range, while also keeping enough clusters in each arm to permit the intended analysis method.

## 1 Background

In individually randomized trials it has long been known that it is more efficient to randomize to arms unequally when the variance of the outcome differs between arms.^1^ In cluster randomized trials unequal randomization is also more efficient when the intracluster correlation (ICC) or the cluster size differs between arms. An expression is available to identify the optimal allocation ratio in this case, for given cluster sizes in each arm.^2,3^ A related expression for individually randomized trials where only one arm is clustered due to treatment has been derived,^4^ but this setting is not considered further in this article. Besides consideration of efficiency, unequal allocation in trials may be chosen because the intervention is more expensive to provide and/or there is a fixed amount of equipment or staff available to provide the intervention. Fewer clusters were allocated to the intervention in the PARAMEDIC trial that randomized ambulances where the intervention was provision of a mechanical compression device for cardiac arrest.^5^ In the ROPE trial, where clusters were villages, exactly 26 villages were allocated to the intervention because there were resources available to train and support 26 teachers to deliver the intervention, one per village.^6^ A potential reason to conversely allocate more clusters (or individuals) to the intervention arm is to improve learning about its implementation.

In many cluster randomized trials it is possible for the ICC and variance to differ between arms due to the effect of the intervention(s). We note this can in particular be expected where an intervention standardizes care, for example, through use of an algorithm in medical care, or a device as in the ongoing PARAMEDIC trial.^5^ Such an intervention could cause the ICC and/or variance to decline relative to current care. Conversely interventions may potentially increase the ICC and/or variance if they are not fully implemented in all clusters, or involve adaptation to each cluster such as participatory learning and action interventions that develop local solutions to local challenges as in the ongoing SNEHA-TARA and JIAH trials.^7,8^

In contrast to earlier research,^2,3^ we here investigate optimal design in which not only the allocation of clusters is allowed to vary but also the allocation of individuals to be measured to the trial arms. In other words while we assume a common cluster size within trial arms the cluster size is allowed to differ between arms and is selected freely. Hemming et al present methodology for sample size calculation with different ICC and cluster size between arms.^9^ In some trials the number of individuals recruited or exposed per cluster is determined by the setting, for example, because the intervention is delivered at the level of a health facility and all individuals in the local area are affected. Nevertheless our methodology may still be helpful in this case because it allows trialists to see how to minimize the number of individuals that need to be measured in the trial, where outcomes are not routinely collected and only a random sample of those exposed are measured. Such a design is common in community randomized trials where the number of individuals exposed per cluster may be very large as in the SNEHA-TARA trial.^7^ We use the term *cluster size* in this article to denote the number of individuals measured in a cluster, and we assume all clusters provide some measurements. Identifying an optimal design, meaning one with the minimum number of individuals measured for a given number if clusters, is particularly important if the data collection is burdensome or expensive, such as in the PA4E1 school randomized trial where the student-level data collection includes 7-day accelerometer measurements, anthropometry, and a survey.^10^

We restrict our attention in this article to parallel group trials, without baseline measurement, and a continuous outcome. We first establish how to identify the optimal allocation of clusters and individuals measured without constraints, and then when the number of clusters or cluster size is constrained in one or both trial arms. We show how trialists could investigate alternative suboptimal (less efficient) designs which could be nevertheless be preferred for logistical or other reasons. We also provide an approach to considering trial design in practice under uncertainty in the ICC or other values required to calculate sample size. We show how to calculate sample size, and illustrate our approach through considering how the PA4E1 trial might have been designed under a range of hypothetical (but realistic) alternative scenarios leading to unequal allocation.

## 2 Model and Power Function

The data are assumed to follow this model:

$$Y_{ij}\;=\;\beta_{0}\;+\;\beta_{1}X_{i}\;+\;(1\;-\;X_{i})u_{0i}\;+\;X_{i}u_{1i}\;+(1\;-\;X_{i})\varepsilon_{0ij}\;+\;X_{i}\varepsilon_{1ij}$$

for participant *j* in cluster *i*, where *X_i_* denotes the trial arm for cluster *i* (coded 0 for control and 1 for intervention). We assume the random terms are Normally distributed with mean zero, but with potentially different variance according to the trial arm. The individual variability term is denoted *ε*_0*ij*_ or *ε*_1*ij*_ depending on trial arm and is assumed independent of the cluster random effect denoted *u*_0*i*_ or *u*_1*i*_. We denote the variance of the random terms thus

$$Var(\varepsilon_{0ij})\;=\;\sigma_{0\varepsilon }^{2};Var(\varepsilon_{1ij})\;=\;\sigma_{1\varepsilon }^{2};Var(u_{0i})\;=\;\sigma_{0u}^{2};Var(u_{1i})\;=\;\sigma_{1u}^{2}$$

and from these we can define the intracluster correlation (ICC) for each arm:

(1) $$\rho_{0}\;=\;\frac{\sigma_{0u}^{2}}{\sigma_{0u}^{2}+\sigma_{0\varepsilon }^{2}};\rho_{1}\;=\;\frac{\sigma_{1u}^{2}}{\sigma_{1u}^{2}+\sigma_{1\varepsilon }^{2}}.$$

We denote the overall variance of *Y* by $\sigma_{0}^{2}\;=\;Var(Y_{ij}|X_{i}\;=\;0)$ and $\sigma_{1}^{2}\;=\;Var(Y_{ij}|X_{i}\;=\;1)$ and denote their ratio by $\gamma \;=\;\sigma_{1}^{2}/\sigma_{0}^{2}.$

To describe different designs we denote the total number of individuals measured by *N*, and the total number of clusters randomized (we assume all contribute measurements) by *K*. We assume a common cluster size of *m*_0_ for clusters in the control arm and *m*_1_ in the intervention arm. We denote the proportion of measured individuals in the intervention arm by *p* and the proportion of trial clusters allocated to the intervention arm by *g*. When design choice is unconstrained we consider designs across all possible values of *p* and *g* between 0 and 1, given values of *N* and *K*. These in turn define the cluster sizes because

(2) $$m_{1}\;=\;\frac{pN}{gK};m_{0}\;=\;\frac{(1-p)N}{(1-g)K}.$$

In order to consider power we define the target difference in *Y* to detect as *d*. We define the power function, based on a Normal distribution for large sample sizes, thus:

(3) $$\left(\frac{d}{\sigma_{0}\sqrt{\frac{1+(m_{0}-1)\rho_{0}}{(1-p)N}+\frac{\gamma [1+(m_{1}-1)\rho_{1}]}{pN}}}-Z_{1-\frac{\alpha }{2}}\right)$$

Noting that 1 + (*m*_0_ – 1)*ρ*_0_ and 1 + (*m*_1_ – 1)*ρ*_1_ take the form of the design effect commonly used in sample size calculations, we can define the arm-specific effective sample size (ESS) for the two trial arms in terms of either *p* and *N* or *g* and *K* as

(4) $$ESS_{0}\;=\;\frac{(1-p)N}{1+(m_{0}-1)\rho_{0}}\;=\;\frac{(1-g)Km_{0}}{1+(m_{0}-1)\rho_{0}};\;ESS_{1}\;=\;\frac{pN}{1+(m_{1}-1)\rho_{1}}\;=\;\frac{gKm_{1}}{1+(m_{1}-1)\rho_{1}}$$

and hence can alternatively write the power function as

(5) $$\left(\frac{d}{\sigma_{0}\sqrt{\frac{1}{ESS_{0}}+\frac{\gamma }{ESS_{1}}}}-Z_{1-\frac{\alpha }{2}}\right)$$

From this we see that power is maximized at the minimum value of

(6) $$\frac{1}{ESS_{0}}+\frac{\gamma }{ESS_{1}}.$$

## 3 Optimal Design Given Different Constraints

### 3.1 I Unconstrained design

First we investigate how, for given values of *N* and *K*, power can be maximized over *p* and *g*, without constraints on their values.

Based on differentiation of Equation (6), further details provided in the Appendix, we find power is maximized when

$$p_{opt\;}\;=\;\frac{\sqrt{\gamma (1-\rho_{1})}}{\sqrt{\gamma (1-\rho_{1})}+\sqrt{1-\rho_{0}}};g_{opt}\;=\;\frac{\sqrt{\gamma \rho_{1}}}{\sqrt{\rho_{0}}+\sqrt{\gamma \rho_{1}}},$$

and the corresponding optimal allocation ratios are

(7) $$\frac{p_{opt\;}}{1-p_{opt\;}}\;=\;\sqrt{\frac{\gamma (1-\rho_{1})}{1-\rho_{0}}},$$

(8) $$\frac{g_{opt}}{1-g_{opt}}\;=\;\sqrt{\frac{\gamma \rho_{1}}{\rho_{0}}}.$$

Recall from Equation (1) that the ICC is the proportion of total variability due to variability between clusters. Examining the form of these ratios in Equations (7) and (8) we see the optimal allocation of individuals to arms is proportional to the square root of their within cluster variability, and of clusters is proportional to the square root of their between cluster variability. Considering equal variance by arm as a special case, that is *γ* = 1, power is maximized when

$$p_{opt\;}\;=\;\frac{\sqrt{\left(1-\rho_{1}\right)}}{\sqrt{\left(1-\rho_{1}\right)}+\sqrt{1-\rho_{0}}};g_{opt}\;=\;\frac{\sqrt{\rho_{1}}}{\sqrt{\rho_{0}}+\sqrt{\rho_{1}}}$$

and if furthermore *ρ*_1_ = *ρ*_0_ then *p_opt_* = *g_opt_* = 1/2.

We see the optimal design allocates more clusters to intervention than control (ie, *g_opt_* > 0.5) if and only if *γρ*_1_ > *ρ*_0_, and fewer than half the individuals measured (*p_opt_* < 0.5) if and only if (1 – *ρ*_0_) > *γ*(1 – *ρ*_1_). If the two ICCs diverge, for example, *ρ*_1_ → 1 and *ρ*_0_ → 0, then progressively more of the clusters and fewer of the individuals are allocated to the arm with the higher ICC. This is intuitive because as the ICC tends to zero the clustering of the data becomes irrelevant and a few large clusters are as informative as many small ones, conversely as the ICC tends to 1 then many clusters are required and the size of these becomes unimportant.^11^

### 3.2 Design with a fixed allocated proportion of clusters or measurements to arms

The expressions for *p_opt_* and *g_opt_* for an unconstrained design involve only *γ, ρ*_0_, and *ρ*_1_. This indicates that for given values of *N* and *K* power is maximized at *p* = *p_opt_* whatever the value of *g* and at *g* = *g_opt_* whatever the value of *p*.

### 3.3 I Design with fixed cluster size in one arm only

In Sections 3.3 and 3.4 we investigate how to maximize power, for given values of *N* and *K*, under constraints concerning cluster size. These constraints do not fix a single value for either *p* or *g* as in Section 3.2 but specify a relationship between *p* and *g* such that we need only vary one to identify the optimal design.

We suppose that the cluster size in the intervention arm (*m*_1_) will be fixed, though the methodology can be adapted to either arm. With cluster size fixed in one arm then we need only consider varying one allocation proportion, we choose to vary *g*. We note that we can write

(9) $$m_{0}\;=\;\frac{N-gm_{1}K}{(1-g)K}$$

and substitution for *m*_0_ in the expression for *ESS*_0_in Equation (4) can lead to a power function in *N, K*, and *m*_1_ as in Equation (5).

There is a restriction on the range for *g* given *N* and *K* because the number of measurements in the intervention must be less than *N*, that is, *gKm*_1_ < *N*. Deriving an expression for *g_opt_* is challenging and importantly, unlike our other design scenarios, it will depend on both *N* and *K*.

### 3.4 Design with fixed cluster size for each arm

We finally consider optimal design conditional on cluster size in the two arms, *m*_0_, *m*_1_. This scenario has been addressed in the literature,^2,3^ but is included here for completeness and to clarify how this differs from the other scenarios we have considered in Sections 3.1 to 3.3. Power is maximized at the value of the individual allocation ratio given by

$$\frac{p_{opt\;}}{1-p_{opt\;}}\;=\;\sqrt{\frac{\gamma [1+(m_{1}-1)\rho_{1}]}{1+(m_{0}-1)\rho_{0}}}.$$

From Equation (2) we see

$$\frac{g}{1-g}\;=\;\frac{m_{0}p}{m_{1}(1-p)}.$$

So for fixed cluster sizes for each arm the optimal allocation ratio can equivalently be expressed as a cluster allocation ratio thus

$$\frac{g_{opt}}{1-g_{opt}}\;=\;\frac{m_{0}}{m_{1}}\sqrt{\frac{\gamma [1+(m_{1}-1)\rho_{1}]}{1+(m_{0}-1)\rho_{0}}}.$$

## 4 Sample Size Formulas

From Equation (3) we see that a large sample equation for the sample size (here considered both *N* and *K* jointly) is given by

$$\frac{1+(m_{0}-1)\rho_{0}}{\left(1-p\right)}\;+\;\frac{\gamma [1+(m_{1}-1)\rho_{1}]}{p}\;=\;\frac{Nd^{2}}{\sigma_{0}^{2}{\left(Z_{1-\frac{\alpha }{2}}+Z_{1-\beta }\right)}^{2}},$$

where 1 - *β* indicates the desired power. Expressions for *m*_0_ and *m*_1_ can be substituted, based on *N, K, p*, and *g*, following Equation (2), and after some rearrangement we see

(10) $$N[\frac{d^{2}}{\sigma_{0}^{2}{\left(Z_{1-\frac{\alpha }{2}}+Z_{1-\beta }\right)}^{2}}-\frac{\gamma \rho_{1}}{gK}-\frac{\rho_{0}}{(1-g)K}]\;=\;\frac{1-\rho_{0}}{\left(1-p\right)}+\frac{\gamma (1-\rho_{1})}{p}.$$

This has a valid solution (*N* > 0) provided the expression in square brackets is positive, and therefore

$$K>\frac{\sigma_{0}^{2}{\left(Z_{1-\frac{\alpha }{2}}+Z_{1-\beta }\right)}^{2}}{d^{2}}[\frac{\gamma \rho_{1}}{g}+\frac{\rho_{0}}{\left(1-g\right)}].$$

Equation (10) can be used in any of the scenarios considered across Sections 3.1 to 3.4, but where there are constraints on cluster size as in Sections 3.3 and 3.4 these will impose a relationship between *p* and *g* that must be incorporated. To consider sample size for optimal unconstrained designs (Section 3.1), expressions for *p_opt_* and *g_opt_*, in terms of *γ,ρ*_0_, and *ρ*_1_, can be inserted for *p* and *g*. When *p* or *g* is fixed as in Section 3.2 its value can be easily inserted into (10). A special case is where we consider a fixed *g* as in Section 3.2, and investigate *N* given *K*, so that the numbers of clusters in each arm (*K*_0_ and *K*_1_) are fixed. Equation (10) can be re-expressed for this case to calculate *N* only thus

(11) $$N[\frac{d^{2}}{\sigma_{0}^{2}{\left(Z_{1-\frac{\alpha }{2}}+Z_{1-\beta }\right)}^{2}}-\frac{\gamma \rho_{1}}{K_{1}}-\frac{\rho_{0}}{K_{0}}]\;=\;\frac{1-\rho_{0}}{\left(1-p\right)}+\frac{\gamma (1-\rho_{1})}{p}.$$

## 5 Calculating Sample Size For Optimal Designs When Icc and Variance are Known: Examples Based Around the PA4E1 Trial

The formulas in the previous section allow designs with different pairs of *N* and *K* to be identified that will provide the desired power. To search for suitable designs it can be convenient to choose a range of values for either *N* or *K* and for each value solve for the other. In most settings it will be more appropriate to select a range of values for *K* because the number of clusters that can be included in the trial is often within a particular range, for logistical or cost reasons. There is also a lower bound for *K* at which even if *N* approaches infinity the desired power cannot be attained, as demonstrated by the inequalities in *K* we presented in the previous section.

The ongoing Physical Activity for Everyone (PA4E1) school randomized trial in Australia assesses the effectiveness of an intervention to promote physical activity in school students compared with routine practice. The primary outcome is assessed at the school level, but important outcomes are assessed at the student level. Because of the measurement burden and cost the student outcomes are collected from only a subset of students (40) within each of a subset of schools (*K* = 30 in total, 15 per arm) nested within the trial (total 76 schools, 38 per arm). In total then outcomes will be obtained from N = 1200 students. We focus on the outcome minutes of physical activity within school hours per day, which is measured over a week based on each student wearing an accelerometer. No basis of the sample size for the nested student level data collection is provided in the published protocol,^10^ but we can calculate that hypothetically assuming an ICC of 0.05 in both arms and equal variance then the design provides 80% power to detect a standardized effect of 0.278. We assume that this power for this effect is desired, and look into alternative designs that could have been chosen under different hypothetical assumptions about the ICC and variance by arm, with and without constraints. We imagine it has been decided to minimize *N* due to the measurement burden, and therefore consider a range of values for *K* from 30 upward to 50.

### 5.1 Unconstrained design

We assume that routine school practice is highly variable, and that the intervention will be implemented fully in all intervention arm schools, thereby standardizing practice and reducing the ICC. We also assume that a high ICC value is possible in the control arm, because activity is largely determined by the school with only modest variability between students. We therefore consider the design of trial assuming the ICC is *ρ*_0_ = 0.1 in control arm and *ρ*_1_ = 0.01 in the intervention arm. The optimum allocation proportions are *p_opt_* = 0.512 and *g_opt_* = 0.240.

The optimal cluster size and number of clusters by arm are displayed in Table 1. In the column headed *N* we report the number of individuals measured implied by *K*_1_, *K*_0_, *m*_1_, and *m*_0_. We present the values of *K*_1_ and *K*_0_ implied by *g_opt_* after rounding to the nearest integer, and then based on these calculate a provisional value of *N* from Equation (11) with which to calculate *m*_1_ and *m*_0_ which are then rounded upward to the next integer. Because of this rounding the value of N displayed is therefore greater than the term N calculated directly in Equation (10). In the final column for comparison we report the number of individuals measured calculated under the constraint that the numbers of clusters and cluster size are equal between arms and again after rounding to integers. The optimal design provides a substantial reduction in the number of individuals, particularly for a smaller number of clusters.

### 5.2 Constrained by a minimum number of clusters in each arm

Small numbers of clusters per arm can lead to a greater chance of imbalance and also invalidate certain common methods of analysis.^12–14^ We may therefore choose to optimize the design subject to a minimum of say 10 clusters per arm. From Table 1 we see that therefore we must revise the design for *K* between 30 and 38, setting *K*_1_ = 10, and the results are presented in Table 2. As expected this constraint increases the number of individuals for a given *K* compared with the unconstrained design but the increase is generally modest and there remains a substantial reduction in the number of individuals compared with a design with equal cluster size by arm and equal allocation of clusters.

### 5.3 Constrained also by a maximum cluster size

**Figure 1:**
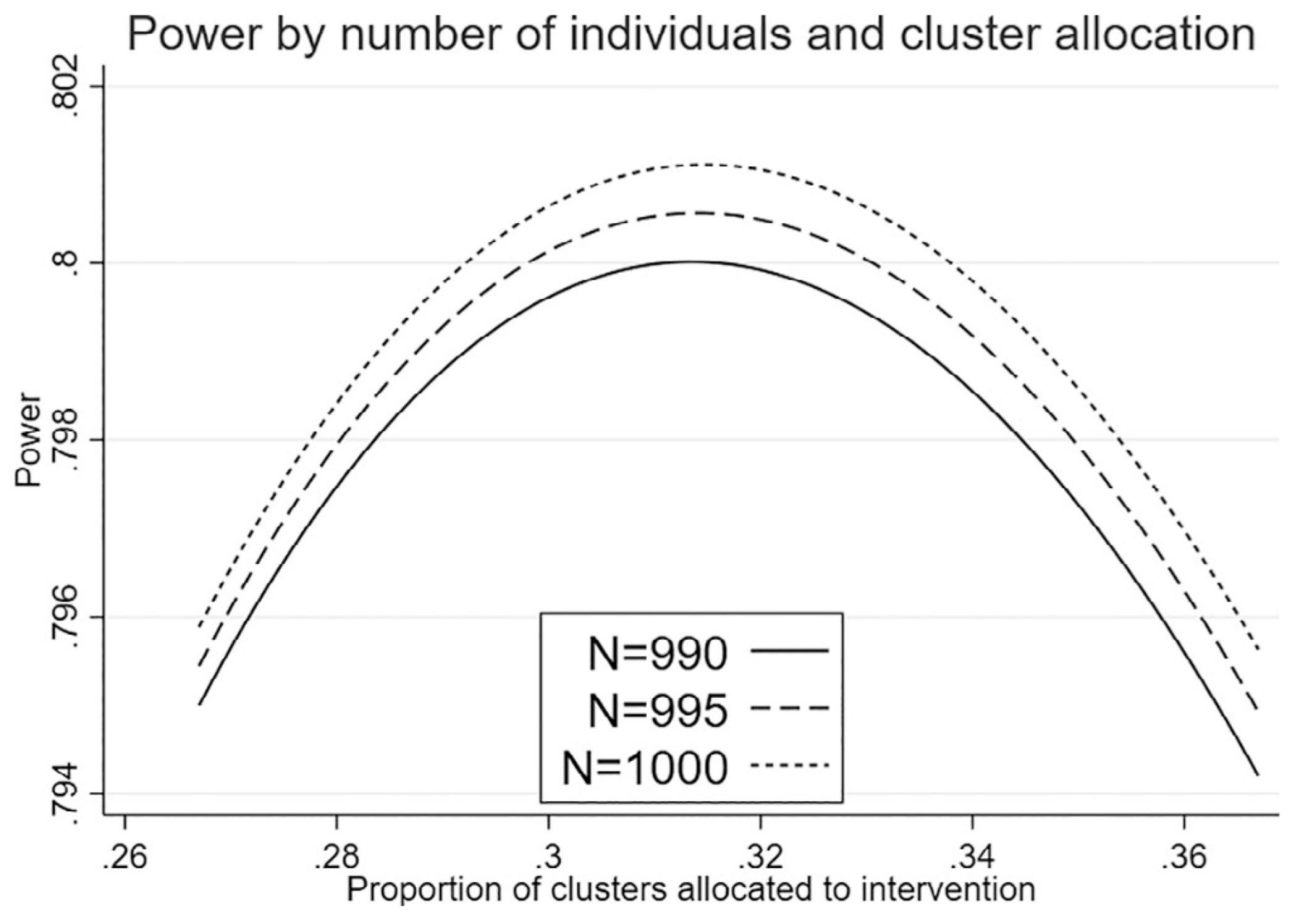
Power for a total of *K* = 30 clusters as *g* and *N* vary, for a fixed cluster size in one arm (*m*_1_ = 45), effect size *d* = 0.278, 5% significance level, and ICCs *ρ*_0_ = 0.1, *ρ*_1_ = 0.01

Here we suppose that the expected maximum feasible size for each cluster is 45 because of concerns over the proportion of students likely to consent to data collection. Considering designs with at least 10 clusters per arm, we see in Table 2 that the optimal design for *K* = 30 is not feasible. We now identify the optimal design for *K* = 30 while setting *m*_1_ to 45. Identifying *g_opt_* algebraically is challenging, so we propose to investigate the design choice and total sample size through an iterative graphical method. For a given value of *K* this involves plotting power curves for increasing values of *N* over the feasible range of values for g to identify the smallest *N* and corresponding value of *g* that achieve the desired power. For each value of *K* and *N*, *g* is constrained by *gKm*_1_ < *N*, though in this example only values of *g* much lower than the constrant are considered given the desired power. Power is calculated from Equation (3) with *m*_1_ = 45 and substituting the expression for *m*_0_ given in Equation (9). The power curves for *K* = 30 are shown in Figure 1 and indicate that in principle N = 990 can provide 80% power, but this is at a value of *g* that cannot be chosen given *K* = 30. If we consider the feasible values of *g* = 0.30 and *g* = 0.33 corresponding to *K*_1_ = 9 and *K*_1_ = 10 respectively we see in both cases 80% power is achieved in principle at N = 995. Respecting the constraint on the number of clusters by arm, we see the optimal design sets *K*_1_ = 10 and *K*_0_ = 20, while *m*_1_ = 45 and (after rounding up) *m*_0_ = 28 and so the total number of measurements in practice is N = 1010. This further constraint has increased *N* by only 20, compared with N = 990 seen in Table 2.

### 5.4 Investigating suboptimal designs

Trialists may wish to also investigate “suboptimal” designs (choices of *p* and *g*) for logistical or other reasons. If for example there are additional costs or resources associated with allocating clusters to the intervention arm then a lower value of *g* than *g_opt_* may be selected if this causes only a modest increase in *N*, the number of individuals to be measured. In the PA4E1 trial this is perhaps unlikely but we expand on our example to illustrate our methodology.

**Figure 2:**
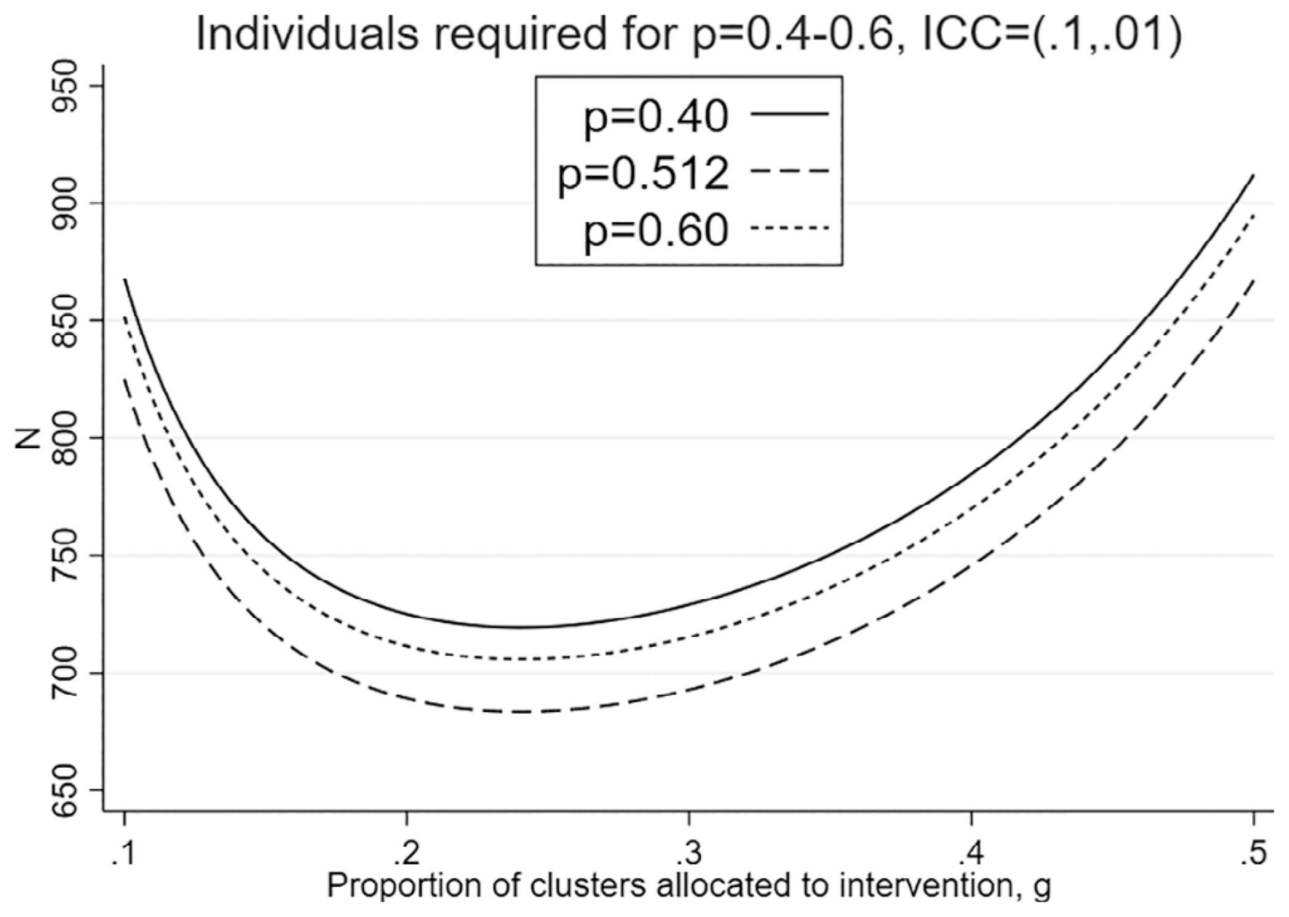
Number of individuals measured, *N*, as *p* and *g* vary from optimal: *K* = 40 clusters, 80% power, 5% significance level, effect size *d* = 0.278, and ICCs *ρ*_0_ = 0.1, *ρ*_1_ = 0.01

We illustrate a graphical approach to investigate suboptimal designs by extending the unconstrained design presented in the previous section, for *K* = 40 as in the seventh row of Table 1. Figure 2 shows the impact on *N* of varying either *p* or *g* away from their optimal values *p_opt_* = 0.512 and *g_opt_* = 0.240. The values of *N* displayed are theoretical number of individuals from Equation (10), in practice *N* will need to be higher due to rounding both cluster size and number of clusters to be integers for each arm. We see that retaining *p* = *p_opt_* = 0.512 but increasing *g* from *g_opt_* (in practice this is *g* = 0.25, *K*_1_ = 10 and *K*_0_ = 30) to 0.40 (*K*_1_ = 16 and *K*_0_ = 24) increases *N* by around 70. We also see that while holding *g* at *g_opt_*, reducing *p* from *p_opt_* to 0.4 increases *N* by around 40, and increasing *p* from *p_opt_* to 0.6 increases *N* by around 25.

## 6 Optimal Designs Under Uncertainty In Icc and Variance

We have previously focused on scenarios where researchers are happy to select single values for the ICC and variance in each trial arm, but uncertainty in these parameters is common in practice and could be expected for settings such as the PA4E1 trial. In this section we address optimal design where the parameters are considered to lie in plausible ranges. We seek to identify the design which, for a given number of clusters, requires the smallest number of individuals measured to achieve a specified power across all values in the ranges.

**Figure 3:**
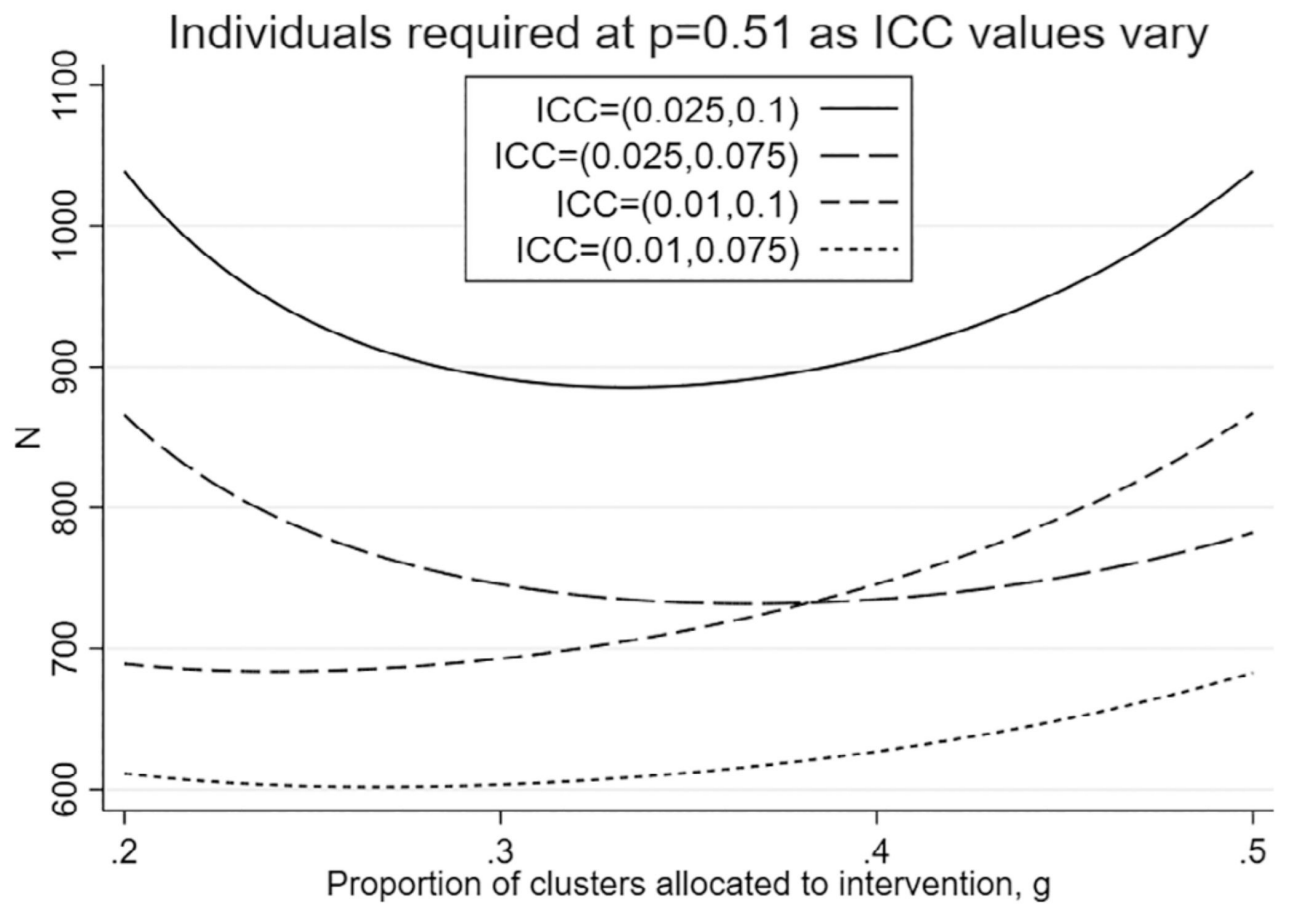
Number of individuals measured, *N*, for different ICC values and varying *g*: *K* = 40 clusters, 80% power, effect size *d* = 0.278, and *p* = 0.51

We next extend our example based on the PA4E1 trial (unconstrained design with *K* = 40) to consider how best to design a trial if there is uncertainty in the values of ICC expected for each trial arm, as there could well be in the PA4E1 trial. We assume *ρ*_0_ lies between 0.075 and 0.1 and *ρ*_1_ lies between 0.01 and 0.025. In Figure 3 we plot how*N* varies with *g* at the four combinations of these boundary values, while holding *p* = 0.51 which is very close to *p_opt_* for every combination (*p_opt_* varies between 0.507 and 0.512). The values of *N* are derived from Equation (10). We see that as expected *N* is larger for larger values of the ICC. While a total sample size of around 750 together with *g* in the range 0.3 to 0.4 would provide adequate power in three of the four scenarios considered, at the extreme where both ICCs are the largest considered, that is, (*ρ*_0_, *ρ*_1_) is (0.1, 0.025) the required sample size increases to at least 880. Choosing *N* = 900 and setting *g* to 0.325 or 0.350 (*K*_1_ = 13 *or* 14) is a conservative choice proving power across all values of the ICC considered.

Thinking more generally it is intuitive that when considering a range for the ICC and/or variance within each trial arm that the design that requires the smallest number of individuals measured given a total number of clusters is the optimal design for the highest values of the parameters (above (0.1, 0.025) for (*ρ*_0_, *ρ*_1_)). This is because any design that achieves the desired power (say 80%) at the highest parameter values will have higher power should their values be lower, and by definition of optimality no alternative design can achieve the desired power with fewer individuals at the highest parameter values. Some further issues around trial design in practice under uncertainty are however discussed in the next section.

## 7 Discussion

Our research has revealed important novel and simple findings concerning optimal trial design where cluster size can be chosen freely by the researchers, identifying designs with unequal allocation when the ICC or variance differ between arms. A striking finding from our examples is how the optimal allocation of individuals measured to arms is very close to equal when ICCs differ to realistic degrees if the variance of the outcome is equal between study arms. More generally from Equation (7) if the ratio of variance for intervention to control is *γ* then the optimal allocation ratio of individuals to the intervention arm relative to control will be very close to $\sqrt{\gamma }.$ For a near optimal design trialists may select these values for their design and then only need investigate the optimal allocation of clusters to arms. We have also described how to identify optimal designs under the sorts of constraints that can arise in practice such as a maximum feasible cluster size,or a minimum number of clusters in each arm. Our hypothetical examples based on the PA4E1 trial demonstrate that appreciable reductions in the number of individuals that need to be measured are possible through the use of optimal designs. As we demonstrated sample size curves can be used to investigate the impact of deviating somewhat from the optimal allocation of clusters or individuals where this is preferred. In many scenarios we expect that some deviation from the optimal design will cause only a modest increase in sample size.

In the design of cluster randomized trials there will often be uncertainty in the ICC and/or variance parameters. This uncertainty is compounded if it is suspected that the intervention itself might affect the ICC and variance. Given plausible ranges for the parameters in each trial arm it is straightforward to design an “optimal” trial that minimizes the number of measurements for a given number of clusters, with at least a specified level of power across all values in the plausible ranges. As discussed in the previous section this optimal design assumes the highest plausible parameter values in each arm. This design may not perform uniformly well over all plausible parameter values, however. Suppose we are confident that the ICC is 0.01 in the control arm but in the intervention arm the plausible range is anything from 0.01 to 0.10 (to keep things simple let us suppose the variance is unaffected by the intervention). The optimal design in this case, based on ICC values of 0.01 and 0.10, allocates clusters in an unbalanced way (*g_opt_* =√10) which would be very inefficient if both ICCs were 0.01. An area of further work is to explore more sophisticated strategies for choosing a design in this case, for example, a Bayesian approach based on priors for the ICC parameters in which we identify sample size to achieve adequate power across ranges for the ICCs well supported by the priors.

An important issue to consider when contemplating design with an unequal cluster size between arms is selection bias. In some settings requiring a larger cluster size will lead to bias as “harder to reach” individuals may be measured, or individuals who only become eligible later, compared with those clusters with a smaller cluster size. This potential for bias may be minimized, where possible, by enumerating all eligible individuals in each cluster, and then selecting at random those to be invited to provide measurements. This approach would be feasible for example in school randomized trials such as PA4E1.

Our findings for an unconstrained design contrast with the literature that has addressed optimal design when the cluster size in each arm is fixed,^2,3^ in which considerably more than half the individuals measured may be in one arm if the ICC is much higher than in the other arm. Our research shows that this however arises only because measuring more individuals given a fixed cluster size will lead to more clusters, it is the greater number of clusters allocated to an arm with a higher ICC that increases the efficiency of the design.

Optimal designs as determined by the large sample formulas we present may not be optimal for small samples, in particular where the number of clusters is modest. Furthermore the methodology we present to calculate sample size requires adaptation when the number of clusters is small.

Our methodology allows trialists to specify a range for the number of clusters in the trial and then for each determine the minimum number of individuals that need to be measured through an optimal design. We do not offer any formal advice as to how the number of clusters should be chosen but we assume that the resource implications of each possible number of clusters can be compared, and “traded off” against the number of individuals required, in order to select the final design. More formal approaches could follow established methods.^3,15^

Besides application to cluster randomized trials, our methodology may also be used to reduce the number of individuals needing to be measured in nonrandomized interventional studies, where an unequal allocation of clusters may be selected and measurement is burdensome. Even if the intervention does not affect the ICC or variance, our methods can be used to show that different cluster sizes in the study arms are optimal. One example where optimal designs could have been considered is a general practice based study of “social prescribing” where individuals provided quality-of-life outcome measures.^16^ Another example is a school-level study of a physical activity and nutrition intervention, where outcomes required pupil questionnaires, accelerometer use, anthropometry, a physical fitness test, and a parent questionnaire outcome measures.^17^

Further work could address optimal design for other outcome types such as binary or count, and also address design when baseline measurements of the outcome are available for either the same individuals measured later or different individuals in the same clusters.

It is difficult to know how often values of the variance and ICC differ between arms because they are infrequently reported separately by arm and the CONSORT guidance does not recommend doing so.^18^ An important area of further research would be to investigate the sorts of trial settings and interventions in which variance or ICC will differ between arms to a degree that should be accounted for in design (and analysis). We recommend that both the variance and ICC should be reported separately by arm as we argue it will be rare that it can be assumed with any certainty that the intervention does not affect these.

## Supplementary Material

Supplementary Appendix

## Figures and Tables

**Table 1:** Optimal unconstrained design and number of individuals measured as total number of clusters *K* varies between 30 and 50 (80% power, *d* = 0.278, *ρ*_0_ = 0.1, *ρ*_1_ = 0.01, *p_opt_* = 0.512, and *g_opt_* = 0.240)

*K*	*K*_0_	*K*_1_	*m*_0_	*m*_1_	*N*	*N_equal_* ^a^
30	23	7	20	68	936	1500
32	24	8	18	55	872	1280
34	26	8	15	51	798	1122
36	27	9	14	43	765	1044
38	29	9	12	41	717	950
40	30	10	12	36	720	880
42	32	10	11	34	692	840
44	33	11	10	30	660	792
46	35	11	9	29	634	782
48	36	12	9	26	636	720
50	38	12	8	26	616	700

a Number of individuals measured if number of clusters and cluster size forced equal between arms.

**Table 2:** Optimal design and number of individuals measured as total number of clusters *K* varies between 30 and 38, constrained to at least 10 clusters in each arm (80% power, *d* = 0.278, *ρ*_0_ = 0.1, *ρ*_1_ = 0.01, *p_opt_* = 0.512, and *g_opt_* is as close to the unconstrained optimal value 0.240 as possible given the constraint)

*K*	*K*_0_	*K*_1_	*m*_0_	*m*_1_	*N*	*N_equal_* ^a^
30	20	10	24	51	990	1500
32	22	10	20	45	890	1280
34	24	10	17	42	828	1122
36	26	10	15	39	780	1044
38	28	10	13	37	734	950

a Number of individuals measured if number of clusters and cluster size forced equal between arms.

## Data Availability

Data sharing is not applicable to this article as no new data were created or analyzed in this study.
