# Supplementary material for: Optimal design of cluster randomized trials allowing unequal allocation of clusters and unequal cluster size between arms
Source: Stat Med. Author manuscript; Available in PMC 2023 Jun 15. (PMC7614658; doi:10.1002/sim.9135)
Supplement: Supplementary Appendix [file EMS176928-supplement-Supplementary_Appendix.pdf]

APPENDIX 1. MAXIMIZATION OF POWER FUNCTION TO IDENTIFY OPTIMAL VALUES OF P AND G

We noted earlier that the power function can be written, in the notation of Section 2, as

$$\Phi\left(\frac{d}{\sigma_0\sqrt{\frac{1}{ESS_0}+\frac{\delta}{ESS_1}}}-Z_{1-\frac{\alpha}{2}}\right)$$

10970258, 2021, 25, Downloaded from https://onlinelibrary.wiley.com/doi/10.1002/sim.9135 by University College London UCL Library Services, Wiley Online Library on [07/06/2023]. See the Terms and Conditions (https://onlinelibrary.wiley.com/terms-and-conditions) on Wiley Online Library for rules of use; OA articles are governed by the applicable Creative Commons License

and that from this we see that power is maximized at the minimum value of

$$\frac{1}{ESS_0} + \frac{\delta}{ESS_1}.$$

This expression needs to be re-expressed in terms of p and g to be minimized. The expression can be alternatively written, as seen in Equation (2), as

$$\frac{1 + (m_0 - 1)\rho_0}{(1-p)N} + \frac{\delta[1 + (m_1 - 1)\rho_1]}{pN}.$$

We need next to substitute expressions for m_0 and m_1 given in Equation (1) thus

$$m_1 = \frac{pN}{gK}; m_0 = \frac{(1-p)N}{(1-g)K},$$

and this leads finally to needing to minimize

$$\frac{1 + \left(\frac{(1-p)N}{(1-g)K} - 1\right)\rho_0}{(1-p)N} + \frac{\delta\left[1 + \left(\frac{pN}{gK} - 1\right)\rho_1\right]}{pN}.$$

This minimization was performed in Matlab through partial differentiation with respect to p and setting to zero, and then repeating for g. This was sufficient to find the optimal values of p and g jointly because the expression for p_{opt} does not involve g and vice versa.

APPENDIX 2. STATA CODE TO GENERATE THE SAMPLE SIZE PLOTS IN FIGURES 2 AND 3

Code for Figure 2

k is the total number of clusters (in the article denoted K)
rhoc is the ICC in the control arm (ρ_0)
rhoi is the ICC in the intervention arm (ρ_1)
d is the standardized effect size
the values 0.4, 0.512 and 0.6 are values of the proportion of individuals allocated to the intervention arm (p)
x is the proportion of clusters allocated to the intervention arm (g)
the value 7.84 is chosen for 80% power and 5% significance level $\left(Z_{1-\frac{\alpha}{2}} + Z_{1-\beta}\right)^2$

```
foreach k of num 40 {
foreach rhoc of num 0.1 {
foreach rhoi of num 0.01 {
foreach d of num 0.278 {
twoway function y = (((1-‘rhoc’)/(1-0.4)) + ((1-‘rhoi’)/0.4)) / ((‘d’*‘d’/7.84) - (‘rhoi’/(x*‘k’)) - (‘rhoc’/((1-x)*‘k’))), range(0.1 0.5) ylabel(650(50)950) lcolor(blue) || ///
function y = (((1-‘rhoc’)/(1-0.512)) + ((1-‘rhoi’)/0.512)) / ((‘d’*‘d’/7.84) - (‘rhoi’/(x*‘k’)) - (‘rhoc’/((1-x)*‘k’))), range(0.1 0.5) ylabel(650(50)950) lcolor(purple) || ///
function y = (((1-‘rhoc’)/(1-0.6)) + ((1-‘rhoi’)/0.6)) / ((‘d’*‘d’/7.84) - (‘rhoi’/(x*‘k’)) - (‘rhoc’/((1-x)*‘k’))), range(0.1 0.5) ylabel(650(50)950) lcolor(red) ///
title("measurements required for p=0.4-0.6, ICC=(‘rhoc’,‘rhoi’)") ytitle("N") xtitle("Proportion of clusters allocated to intervention, g") ///
legend(label(1 "p=0.40") label(2 "p=0.512") label(3 "p=0.60") pos(12) ring(0) forcesize symxsize(8) symysize(1) rowgap(1) size(large) colgap(1) symplacement(left)textfirst cols(1) colfirst)
graph export "Figure 2.png", replace
}
}
}
}
```

10970258, 2021, 25, Downloaded from https://onlinelibrary.wiley.com/doi/10.1002/sim.9135 by University College London UCL Library Services, Wiley Online Library on [07/06/2023]. See the Terms and Conditions (https://onlinelibrary.wiley.com/terms-and-conditions) on Wiley Online Library for rules of use; OA articles are governed by the applicable Creative Commons License

Code for Figure 3

k, x and d are defined as for Figure 2, and 7.84 is again chosen for 80% power.

Here the value of p is selected to be 0.51

The different lines are defined by different pairs of (ρ_0, ρ_1), and in the order shown these are (0.1, 0.025), (0.075, 0.025), (0.1, 0.01), (0.075, 0.01)

```
foreach k of num 40 {
foreach d of num 0.278 {
twoway function y = (((1-0.1)/(1-0.51)) + ((1-0.025)/0.51)) / ((‘d’*‘d’/7.84) - (0.025/(x*‘k’)) - (0.1/((1-x)*‘k’))), range(0.2 0.5) ylabel(600(100)1100) lcolor(purple) || ///
function y = (((1-0.075)/(1-0.51)) + ((1-0.025)/0.51)) / ((‘d’*‘d’/7.84) - (0.025/(x*‘k’)) - (0.075/((1-x)*‘k’))), range(0.2 0.5) ylabel(600(100)1100) lcolor(brown) || ///
function y = (((1-0.1)/(1-0.51)) + ((1-0.01)/0.51)) / ((‘d’*‘d’/7.84) - (0.01/(x*‘k’)) - (0.1/((1-x)*‘k’))), range(0.2 0.5) ylabel(600(100)1100) lcolor(red)|| ///
function y = (((1-0.075)/(1-0.51)) + ((1-0.01)/0.51)) / ((‘d’*‘d’/7.84) - (0.01/(x*‘k’)) - (0.075/((1-x)*‘k’))), range(0.2 0.5) ylabel(600(100)1100) lcolor(blue) ///
title("measurements required at p=0.51 as ICC values vary") ytitle("N") xtitle("Proportion of clusters allocated to intervention, g") ///
legend(label(1 "ICC=(0.025, 0.1)") label(2 "ICC=(0.025, 0.075)") label(3 "ICC=(0.01, 0.1)") label (4 "ICC=(0.01, 0.075)") pos(12) ring(0) forcesize symxsize(8) symysize(1) rowgap(1) size(medium) colgap(1) symplacement(left) textfirst cols(1) colfirst)
graph export "Figure 3.png", replace
}
}
```

10970258, 2021, 25, Downloaded from https://onlinelibrary.wiley.com/doi/10.1002/sim.9135 by University College London UCL Library Services, Wiley Online Library on [07/06/2023]. See the Terms and Conditions (https://onlinelibrary.wiley.com/terms-and-conditions) on Wiley Online Library for rules of use; OA articles are governed by the applicable Creative Commons License
